# Densification of a NASICON-Type LATP Electrolyte Sheet by a Cold-Sintering Process

**DOI:** 10.3390/ma14164737

**Published:** 2021-08-22

**Authors:** Naoki Hamao, Yuki Yamaguchi, Koichi Hamamoto

**Affiliations:** Innovative Functional Materials and Research Institute, National Institute of Advanced Industrial Science and Technology (AIST), 2266-98, Shimo-shidami Anagahora, Moriyama-ku, Nagoya 406-8560, Japan; y-yamaguchi@aist.go.jp (Y.Y.); k-hamamoto@aist.go.jp (K.H.)

**Keywords:** Li-ion conductor, solid electrolyte, NASICON-type oxide

## Abstract

A NASICON-type Li_1.3_Al_0.3_Ti_1.7_(PO_4_)_3_ (LATP) electrolyte sheet for all-solid-state batteries was fabricated by a cold sintering process (CSP). The microstructure of the LATP sheet was controlled to improve the wettability which is an essential factor in CSP. The porous sheets of LATP were prepared by calcination the green sheets to remove the organic components and form the porous structure. By the CSP using the porous sheets, the densification of grain boundary was observed and further densified with increasing reaction time. The total conductivity of the prepared LATP sheet was improved from 3.0 × 10^−6^ S/cm to 3.0 × 10^−4^ S/cm due to the formation of necks between the particles at the grain boundary.

## 1. Introduction

All-solid-state batteries that use oxides as solid electrolytes are expected to be among high-performance next-generation energy devices. For the practical application of all-solid-state batteries with high energy density, not only the search for new solid electrolyte materials with high conductivity, but also research on the improvement of the conductive properties of electrolyte materials is attracting attention. Although thin and dense solid electrolytes can reduce a battery’s internal resistance and suppress the dendrite formation, they can also cause the battery to short circuit [1,2,3,4,5,6]. As an inorganic electrolyte material, the NASICON-type Li_1.3_Al_0.3_Ti_1.7_(PO_4_)_3_ (LATP), is a promising electrolyte for oxide all-solid-state batteries due to its excellent chemical stability and high ionic conductivity at room temperature [7,8,9,10]. Although LATP is a glass-ceramic material with a lower glass transition (650 °C) and crystallization temperature (690 °C) than those of other solid electrolyte materials, a temperature of about 1150 °C is still required for its densification without a sintering aid [11]. In the case of the solid electrolytes, two negative effects, lithium loss and second phase formation, can occur during high-temperature sintering. Therefore, the development of low-temperature sintering technology is essential for fabricating solid electrolytes. In recent years, the cold sintering process (CSP) has attracted much attention as a technique to improve the density of ceramics at low temperatures [12,13,14]. CSP improves the density of ceramics at low temperatures by sintering ceramics and metals together with a small amount of water at temperatures below 300 °C and applied pressure (<400 MPa). In general, CSP consists of several processes:

1. Dissolution of ceramics particles from surface into aqueous solution
2. Rearrangement of particles
3. Formation of a supersaturated solution through the evaporation of the aqueous solution
4. Crystal growth and recrystallization of the metastable phase

Recently, the densification of LATP-based solid electrolytes by CSP has been reported in papers [15,16]. Liu et al. investigated the densification of LATP pellets by CSP and reported that the density of LATP electrolytes is strongly related to the sintering process, solvent addition, and applied stress [15]. In addition, in magnetic material systems, Lowum et al. reported the densification of BaFe_12_O_19_ by CSP and investigated the relationship between the amount of solution used in CSP and the sintered material’s microstructure [17]. They observed densification of the BaFe_12_O_19_ particles when an optimal amount of water was applied. However, increasing the amount of water applied past this optimal amount caused the particles to be coated by a secondary material. Moreover, Bouville and Studart [18] studied the densification of agglomerates by CSP. They reported no sintering behavior was observed in the N-methyl pyrrolidone solutions because ion dissolution and liquid-induced plasticity do not occur in organic solvents.

In this study, we incorporated CSP into a two-step sintering process to obtain dense LATP sheets at a low temperature. Thin LATP electrolyte sheets that are difficult to fabricate by conventional CSP using mortar mixed with ceramics powder and water, were fabricated. Then, we attempted to densify thin LATP sheets by CSP and examined the changes in the microstructure and conductivity of LATP.

## 2. Materials and Methods

Li_1.3_Al_0.3_Ti_1.7_(PO_4_)_3_ (LATP) powder was prepared by solid-state reaction method. Li_2_CO_3_ (99.9%, Fujifilm Wako Pure Chemical Industries, Osaka, Japan), TiO_2_ (98.5%, Kanto Chemical, Tokyo, Japan), Al(OH)_3_ (99.9%, Kojundo Chemical Lab. Co., Ltd., Saitama, Japan), SiO_2_ (99.9%, Kojundo Chemical Lab. Co., Ltd., Saitama, Japan), and NH_4_H_2_PO_4_ (99.9%, Fujifilm Wako Pure Chemical Industries, Osaka, Japan) were mixed by ball milling (P6, Fritsch Japan, Kanagawa, Japan) at 500 rpm for 1.5 h. The mixed powder was calcined in an electric furnace at 550 °C for 3 h in air. Then, the calcined powder was pulverized and calcined at 1050 °C for 1 h in air.

LATP electrolyte sheet was prepared by the tape-casting method. The obtained powder was milled using a multi beads shocker (MB3000, YASUI KIKAI, Osaka, Japan) at 1800 rpm for 10 min. Then, LATP slurry was prepared using the precursor powder, toluene(98%, Fujifilm Wako Pure Chemical Industries, Osaka, Japan), n-butanol(98%, Fujifilm Wako Pure Chemical Industries, Osaka, Japan), adipic acid(99%, Fujifilm Wako Pure Chemical Industries, Osaka, Japan), 1,3-Diaminopropane(98%, Fujifilm Wako Pure Chemical Industries, Osaka, Japan), and polyvinyl butyral(99%, Fujifilm Wako Pure Chemical Industries, Osaka, Japan). The green tape was prepared with a single doctor blade on a polyester film, and the solvent was removed at room temperature. The green tape was removed from the supporting polyester film and dried at room temperature. To obtain a porous sheet without organic components, the green tape was calcinated in an electric furnace at 650 °C for 2 h in air.

A 10 mm × 10 mm porous LATP sheet with a thickness of about 70 μm and deionized water (weight ratio = 1:0.3) were placed into a 30-mm-diameter stainless steel mold. Then, the stainless-steel mold was set in a press machine(H300-01, AS ONE Co., Osaka, Japan) pre-heated to 200 °C and was uniaxial pressed immediately at 25 MPa. After CSP, the mold was immediately removed from the press and cooled at room temperature. To study the effect of CSP time on the microstructure and conductivity of LATP sheets, the CSP time was set to 5, 10, and 30 min.

The phase identification of prepared LATP sheets was performed by X-ray diffraction (SmartLab, Rigaku, Tokyo, Japan) using Cu Kα radiation with 2θ range from 10° to 70° at an interval step of 0.02°. The cross-sections of the obtained samples were prepared by Ar ion milling using a cross-section polisher (CP, IB-19510CP, JEOL, Tokyo, Japan). The cross-section morphology of the samples was observed using a field-emission scanning electron microscope (FE-SEM, JSM-6330F, JEOL, Tokyo, Japan) at an acceleration voltage of 10 kV with a working distance of 15 mm. To measure the ionic conductivity, Au electrodes were sputtered on both sides of obtained LATP sheet. Then the ionic conductivity was measured with an impedance analyzer (HP-300, BioLogic, Grenoble, French) at room temperature. The measured frequency was from 0.01 to 10^6^ Hz with a 10-mV AC amplitude.

## 3. Results and Discussion

### 3.1. Wetting Behavior of the LATP Sheet

**Figure 1:**
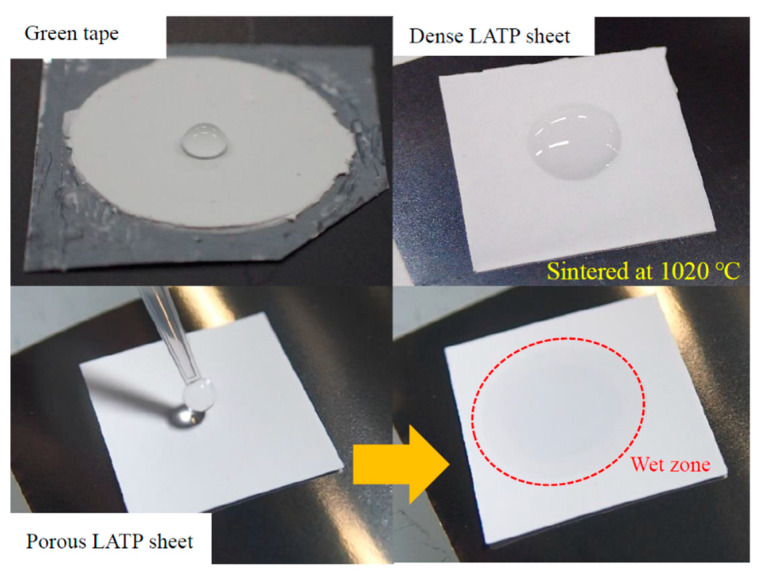
Images of the fabricated LATP electrolyte sheets.

To make sure sintering by CSP proceeds uniformly and quickly, we considered that it is important to supply the optimal amount of water required for the reaction uniformly to the surface of the particles. Figure 1 shows the wetting behaviors of water droplets on LATP sheets with various densities. The green sheet has poor wettability due to its organic components. The dense LATP sheet, prepared at 1020 °C, also shows poor wettability. On the other hand, the porous LATP sheet prepared at 650 °C exhibits good wettability. Based on the observed wetting behaviors of the LATP sheets, we think that the densification effect of CSP could be promoted by sintering LATP sheets with a porous microstructure.

### 3.2. Phase Identification

**Figure 2:**
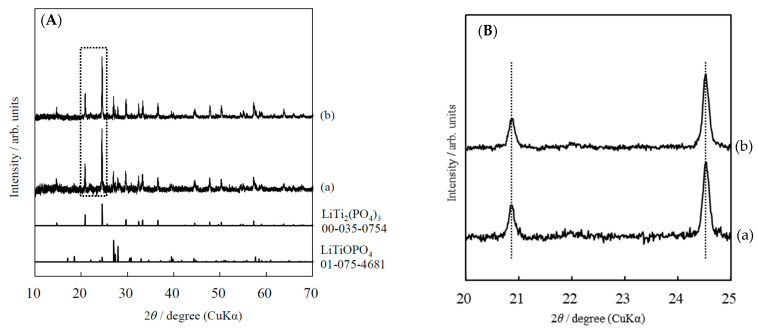
(**A**) XRD patterns of a LATP electrolyte sheets (a) calcined at 650 °C (porous) and (b) CSP with H_2_O 30 wt% at 200 °C for 30 min. (**B**) The enlarged XRD patterns of a LATP electrolyte sheets (a) calcined at 650 °C (porous) and (b) CSP with H_2_O 30 wt% at 200 °C for 30 min from 20 to 25°.

Figure 2A shows the XRD patterns of LATP sheets calcined at 650 °C and CSP-LATP sheets. In the XRD patterns of both sheets, the main peak attributed to the pattern of LiTi_2_(PO_4_)_3_ (JCPDS 00-035-0754) was detected, but the peak attributed to the impurity phase, LiTiOPO_4_ (JCPDS 01-075-04681), was also detected. To avoid Li volatilization during high temperature sintering, the sintering time was shorter than those reported in others [7,8,9]. As a result, an intermediate product phase, LiTiOPO_4_, was observed in the sheets of porous LATP. As shown in Figure 2B, The shift of diffraction peaks of the LATP sheet was not observed by CSP treatment. This indicates that no reaction, such as decomposition or ion exchange reaction, occurred between the LATP and the water added during the CSP.

### 3.3. Cross-Sectional Morphology

**Figure 3:**
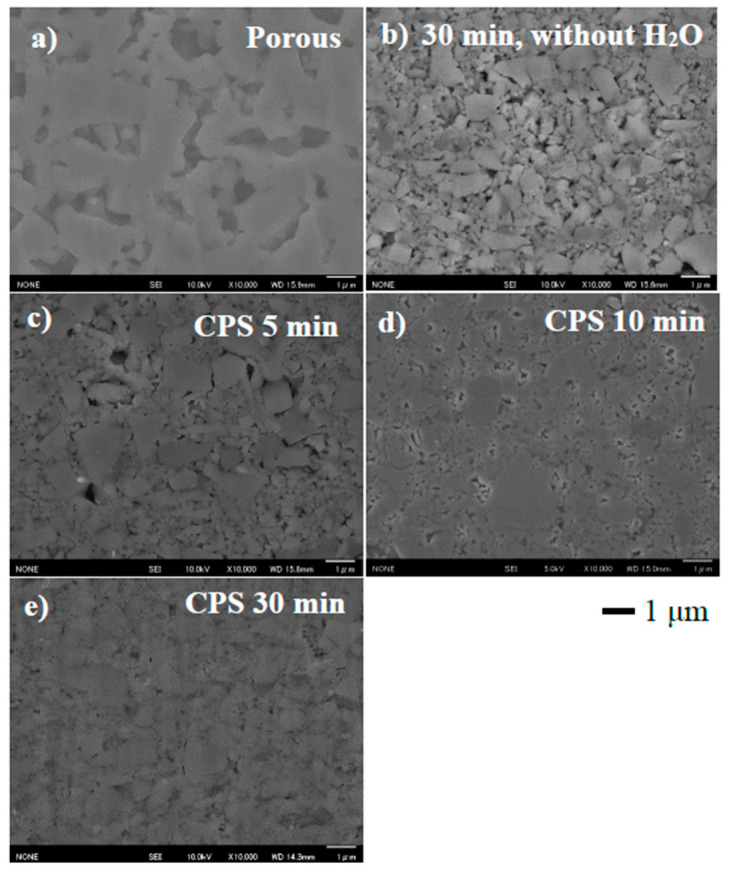
SEM images of the cross-section of LATP electrolyte sheets (**a**) calcined at 650 °C (porous), CSP (**b**) without H_2_O, with H_2_O 30 wt% at 200 °C for (**c**) 5 min, (**d**) 10 min, and (**e**) 30 min.

To investigate the effects the added water and CSP time have on the microstructure of LATP electrolyte sheets, FE-SEM was carried out to observe the cross-sectional morphology of the LATP sheets. Figure 3 shows the SEM images of the cross-section of the porous LATP sheet, the cold-sintered LATP sheets at 200 °C with various CSP times, and the cold-sintered LATP sheet without H_2_O. The thickness of the LATP sheet after the CSP was about 70 μm. The thickness of the sheet did not change significantly with the CSP. In the cross-section of the porous LATP sheet in Figure 3a, the average grain size is 1–5 μm, and the grains are in good contact with each other due to a melting grain-boundary. In the cross-section of the cold sintered LATP sheet without H_2_O, particles of several micrometers and less than 1 μm in size were observed. We considered that the pressurization effect of CSP crushed coarse particles into these fine particles of less than 1 μm in size.

Additionally, cracks due to pressurization during CSP were observed in the coarse particles. On the other hand, the microstructure of the LATP sheets (Figure 3c–e) prepared by CSP was different from that of the porous sheet (Figure 3a). In the cross-section of the cold-sintered LATP sheets in Figure 3c–e, particles with sizes less than 1 μm that were crushed during CSP filled the pores between the larger particles. Although the sintering necks could not be observed at the grain boundaries in the cold-sintered LATP without H_2_O (Figure 3b), it was observed that fine particles were bonded to one another in LATP with 30 wt% H_2_O (Figure 3c–e). This indicates that the uniformed addition of water to the porous LATP electrolyte sheets promoted densification during the CSP, resulting in formation of necks between the grains. Despite a CSP time of only 5 min, a noticeable number of fine particles have already precipitated to fill the LATP sheet’s pores. With increasing CSP time, the amount of the precipitated fine particles increased, and the pores in the LATP sheets were almost filled after 30 min of CSP, as shown in Figure 3e. This led us to conclude that the porosity of the LATP sheets can be reduced with increasing CSP time. 

### 3.4. Electrical Conductivity Measurement

**Figure 4:**
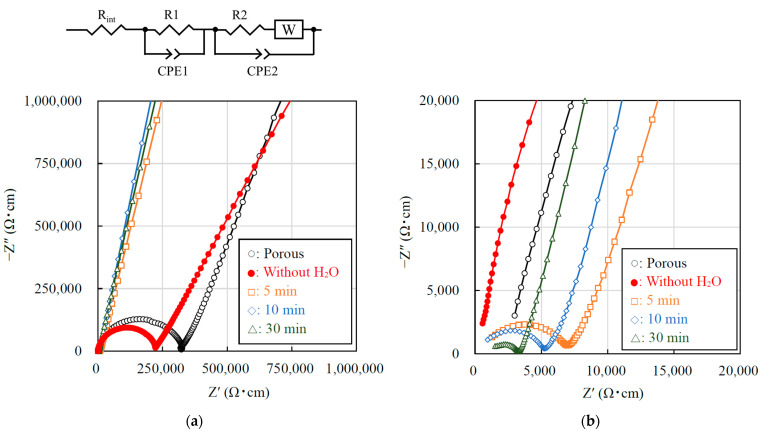
(**a**) Nyquist plots of LATP electrolyte sheets calcined at 650 °C (porous), CSP with H_2_O 30 wt% at 200 °C for 5 min, 10 min, 30 min, and CSP without H_2_O at 200 °C for 30 min. (**b**) Zoomed in the Nyquist plots. ○: Porous, ●: Without H_2_O, □: 5 min, ◇: 10 min, △: 30 min.

**Figure 5:**
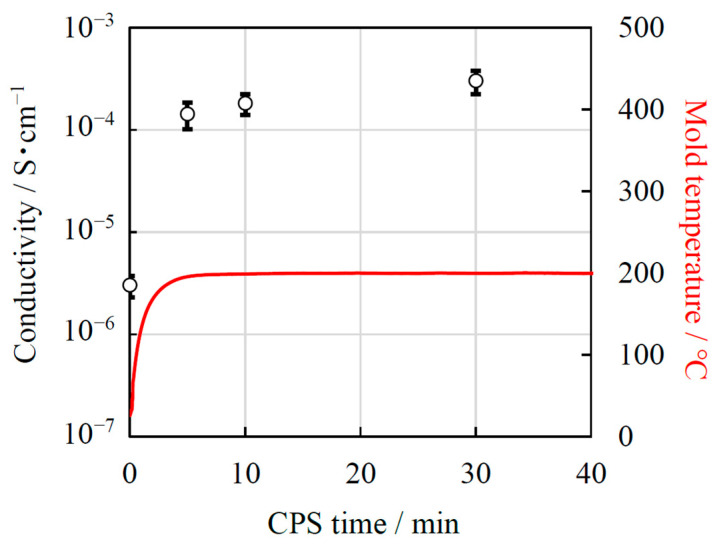
Conductivity of LATP sheets at 25 °C obtained from impedance measurements and mold temperature as functions of CSP time.

To investigate the effects the added water and CSP time have on the conductivity of the LATP sheet, the conductivity measurement was performed Figure 4 shows the Nyquist plots of the porous and cold-sintered LATP sheets at room temperature. In each of the Nyquist plots of all five samples, one semicircle on the high-frequency side and a tail attributed to electrode diffusion on the low-frequency side were observed. In general, in the Nyquist plot of an electrolyte, two semicircles attributed to bulk resistance and grain boundary resistance can be observed on the high and low-frequency sides, respectively. In the present work, the semicircle in Figure 4 was attributed to the total resistance, the sum of the bulk and grain boundary resistance, since the constituent resistances could not be separated. The data were fitted with a series of two parallel combinations of R, CPE, and W circuits, where R corresponds to an ohmic resistor, CPE to a constant-phase element and W represents Warburg impedance. An additional resistance element (with resistance R_int_) in series was also included to account for inductive effects, resulting in the deformation of the high frequency semicircle. The values of the total conductivity of the porous LATP and the cold-sintered LATP without H_2_O were, calculated from the resistances of the high frequency equivalent circuits, 3.0 × 10^−6^ S/cm and 4.4 × 10^−6^ S/cm at 25 °C, respectively. The total conductivity of the porous LATP sheet is lower than that of the conventionally sintered LATP (10^−4^ S/cm) [7]. We attributed this reduction in conductivity to the presence of pores in the electrolyte sheet due to a lower sintering temperature, which was observed in the cross-sectional morphology analysis in Figure 3a. The conductivity of the cold-sintered LATP without H_2_O is slightly improved compared to that of the porous LATP sheet. We attributed this reduction in the total resistance to the decrease in grain-boundary resistance due to the pores being filled by pressurization during CSP. On the other hand, the total conductivities of the LATP sheets prepared by CSP for 5, 10, and 30 min were calculated from the resistances of the high frequency equivalent circuits, 1.4 × 10^−4^, 1.8 × 10^−4^, 3.0 × 10^−4^ S/cm at 25 °C, respectively. The total resistance of the LATP sheet significantly decreases after CSP with the addition of water and continues to gradually decrease with increasing CSP time. We considered that the grain-boundary resistance decreased due to improved interfacial bonding between the particles with increasing CSP time. Figure 5 shows the conductivity of the LATP sheets and the temperature of the stainless-steel mold against CSP time (min). The stainless-steel mold rose to the target temperature of 200 °C after 5 min, which corresponds to the sharp increase in the conductivity of the LATP sheets after CSP. Therefore, we confirmed that this increase in conductivity resulted from the rapid densification of the LATP sheets after the mold and the sample reached 200 °C.

## 4. Conclusions

In summary, dense NASICON-type LATP electrolyte sheets were prepared by a modified CSP using a porous LATP sheet. The conductivity of the porous LATP sheets was improved due to the densification and the formation of a neck between particle at grain boundary by CSP at 200 °C with H_2_O. The grain-boundary resistance decreased with an increase in CSP time, which led to better interfacial bonding among the particles. The ionic conductivity was improved to 3 × 10^−4^ S/cm at a lower pressure and shorter time than previously reported for low-temperature sintering of LATP. The reason for this efficient densification of porous LATP is thought to be that the pressure was uniformly applied to the LATP sheet due to the use of flat LATP sheets. Moreover, the formation of neck at grain boundary can be further promoted by increasing the CSP reaction time. Since no densification of grain boundaries was observed in the LATP sheet after CSP in the absence of water, it is assumed that the small amount of water added to the porous electrolyte was critical to allow densification to take place. We believe that the densification of porous LATP sheets by this simple method will significantly contribute to the production of high-density electrolytes and high-performance batteries for all-solid-state batteries.

## Data Availability

No new data were created or analyzed in this study. Data sharing is not applicable to this article.
